# The Impact of Type 2 Diabetes Mellitus on Long-Term Prognosis in Patients of Different Ages with Myocardial Infarction

**DOI:** 10.1155/2018/1780683

**Published:** 2018-07-10

**Authors:** S. A. Afanasiev, A. A. Garganeeva, E. A. Kuzheleva, A. V. Andriyanova, D. S. Kondratieva, S. V. Popov

**Affiliations:** Cardiology Research Institute, 111a Kievskaya Street, Tomsk 634012, Russia

## Abstract

The objective of the study was to assess the impact of DM2 at baseline on long-term mortality after acute myocardial infarction (MI) among different age groups. The data were taken from: “Register of Acute Myocardial Infarction.” A total of 862 patients were followed for five years after acute myocardial infarction. The primary endpoint was death from any cause. The patients were categorized into 2 groups based on their ages: group 1—comprised patients older than working age (*n* = 358) and group 2—comprised employable patients (*n* = 504). A total of 208 patients were diagnosed with both cardiovascular disease and DM2. Elderly patients with DM2 had worse prognosis and increased five-year mortality compared with patients of the same age group without DM2. Statistically significant differences in long-term outcomes were found in adult patients (*p* = 0.004) only in group with longer duration of diabetes, unlike the group with DM2 onset. In conclusion, Type 2 DM increased 5-year mortality rate of elderly patients with myocardial infarction. However, younger patients with both myocardial infarction and DM2 had more complications in the early post-MI period compared with patients of the same age group without DM2 but did not show any statistically significant differences in the long-term outcome.

## 1. Introduction

Modern achievements in medical science and practice allowed us to obtain tremendous success in the treatment of chronic noncommunicable diseases. Improved prognosis and quality of life in patients suffering from the most common socially significant pathology of the circulatory system—ischemic heart disease—contribute to the progressive aging of population in economically developed countries [1]. One quarter of population of Russian Federation belongs to the category of people who are older than working age (according to Federal State Statistics Service). Such demographic situation determines the increase of the incidence of metabolic disorders; the most common of them is type 2 diabetes mellitus (DM). For the last 10 years, the world number of DM patients has been increased more than twofold and in the end of 2015, had reached 415 million. According to prognosis of the *International Diabetes Federation*, numbers of DM patients will reach about 642 million in 2040. According to data of the Federal Register, in 2016, in Russia, 4 million people were on the dispensary observation for type 2 DM that was almost 3% of the population. However, these data underestimate the real number of patients, as they consider only revealed and registered cases of disease and the real number of diabetes patients in Russia may reach 8-9 million (about 6% of the population) [2, 3].

There is a close link between DM and cardiovascular disease, which is the most prevalent cause of morbidity and mortality in diabetic patients. About 60% of patients are diagnosed with both cardiovascular disease and DM2 [4]. It is known that the risk of development of acute myocardial infarction in patients with type 2 DM is 6–10 times higher than in a whole population [5]. The combination of ischemic heart disease and diabetes is one of the most adverse conditions and leads to the significant increase of cardiovascular complications and the mortality rate.

The problem of comorbidity of these two most common noncommunicable pathologies is traditionally noted in the cohort of patients of elderly and senile age. We are starting to see an increase in type 2 diabetes in leaner people at a much younger age than usually associated with the disease. So about 50% of all patients with type 2 DM in the world are 40–59 years; they are considered to be the working-age population that worsens the economic aspect of this problem [2].

Alongside with that, despite the great interest of the scientific medical community to the problem of comorbidity of ischemic heart disease and type 2 DM, the study of the long-term postinfarction prognosis of patients remains a very complex problem because of necessity to provide optimal number of patients, difficulties of overcoming artificial selectivity of the studied groups, lack of common database of persons who have suffered myocardial infarction, and so on. The use of population registers, such as the Register of Acute Myocardial Infarction, is the most optimal to study the prognostic value of DM in the long-term postinfarction period, because it can ensure the greatest objectivity of the obtained results [6].

In connection with the abovementioned, the aim of this study is to investigate the influence of type 2 diabetes mellitus on long-term prognosis of postinfarction patients in various age categories on the basis of the population taken from Register of Acute Myocardial Infarction.

## 2. Methods

This study was based on analysis of a prospective database “Register of Acute Myocardial Infarction,” Tomsk (Russia). The basis of the information and analytical base of the RAMI is the coding table drawn up based on the specially designed “initial registration record,” which contains all information about a patient (including the results of the interrogation, anamnesis data, medical history data and other medical documents, and the results of pathologist studies). The study included patients with acute myocardial infarction, who were admitted to the Cardiology Research Institute (Tomsk) with acute myocardial infarction within one year (*n* = 862). All the patients included signed the informed consent form; the study protocol was approved by the Local Ethics Committee of the Cardiology Research Institute, Tomsk. The clinical outcome after 5 years was analyzed. To analyze fatal cases from cardiovascular diseases, we have used results of the anatomical-pathological and forensic studies. The study included analysis of five-year mortality depending on age and history of diabetes mellitus 2.

The patients were categorized into 2 groups based on their ages: group 1—comprised patients older than working age (*n* = 358, men over 60, and women over 55 years old) and group 2—comprised employable patients (*n* = 504) (article 7 of the Federal Law from December 17, 2001 number 173-FZ “on labor pensions in the Russian Federation”). The main characteristics of the patients are given in Table 1.

At the admission to the hospital, 208 of the patients had the history of DM2. The diagnosis of diabetes was confirmed according to criteria of the World Health Organization. During the follow-up study, 45 more patients in the second group were found to have DM2 and 33 patients presented the onset of DM2 after discharge from the hospital. Groups were further divided into subgroups according to the duration of diabetes. Patients with diabetes received optimally matched hypoglycemic therapy including glibenclamide, gliclazide, and metformin.

A total of 208 patients were diagnosed with both cardiovascular disease and DM2.

Statistical analyses were performed with Statistica V10.0 (company “StatSoft Inc.” statistical package). Categorical variables were described as frequencies and percentages. Continuous variables were described as mean ± standard deviation (SD). Verification of the quantitative data distribution has been performed using the Shapiro-Wilk test. Qualitative values are presented as absolute and relative values (*n* (%)). To compare quantitative data obeying the normal distribution law in two independent groups, we used Student's *t*-test (homogeneity of general variances has been assessed using the Levene's test). We utilized *χ*^2^ analyses for group comparisons of each of the categorical measurements and the Fisher's test for 2 independent groups for the continuous measurements. The survival analysis in the studied groups was carried out using the Kaplan-Meier method, the comparison of the two curves was performed using the Logrank test. To reveal factors that affect the course and prognosis of the disease, the odds ratio was calculated. The Bonferroni correction was used for multiple comparison correction when several dependent or independent statistical tests were being performed simultaneously. *p* values of 0.05 from two-sided tests were considered to indicate statistical significance.

## 3. Results

### 3.1. Clinical and Anamnestic Characteristics of Patients

In the first group of patients with acute myocardial infarction, there were 158 men older than 60 and 200 women older than 55. In the second group, there were 433 men and 71 women. The groups were divided according to the age of the patients, and they turned out to be different in a number of clinical and anamnestic characteristics.

The older patients suffered from arterial hypertension and angina more often. Every third patient in group 1 had history of myocardial infarction; 12.8% of patients older than working age had stroke history. The majority of patients of working age who suffered myocardial infarction were smokers. The incidence of dyslipidemia in both groups was comparable, and it was 79% and 76.6% of cases.

Every fourth patient in both groups suffered from DM2: 1st group—92 patients (26%) and 2nd group—116 patients (23%) (Table 1). Fasting blood sugar levels in patients of older age were significantly higher than in the group of working-age patients (Table 1).

### 3.2. Survival Rates of Patients after Myocardial Infarction Depending on Age and Diabetes Mellitus

**Figure 1:**
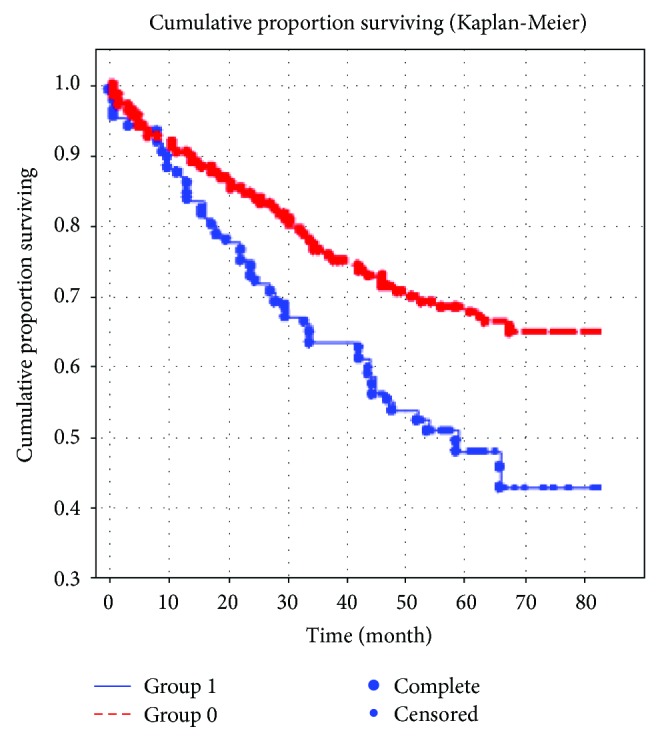
Impact of diabetes mellitus on long-term prognosis of the disease in elderly patients who suffered myocardial infarction. Note: presence of diabetes mellitus: 0 = no; 1 = yes.

The average 5-year mortality rate was 33.8% in group 1 and 26.8% in group 2 (*p* = 0.026). Elderly patients with DM2 had worse prognosis and increased five-year mortality compared with patients of the same age group without DM2 (OD 2.2; 95% CI 1.47–3.4; *p* = 0.002) (Figure 1).

**Figure 2:**
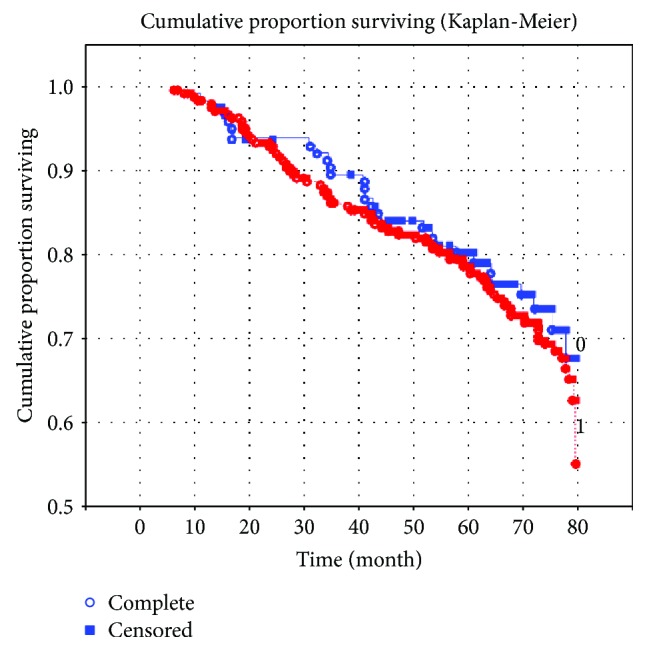
Influence of the presence of diabetes mellitus on long-term prognosis of the disease in patients of working age with myocardial infarction. Note: presence of diabetes mellitus: 0 = no; 1 = yes.

Significant influence of the fact of the presence of type 2 DM on a long-term postinfarction prognosis of the patients has not been revealed in performing similar analysis of survival time of Kaplan-Meier in the cohort of working patients. Thus, the level of 5-year mortality among patients with type 2 DM in this group was 25% (29 persons have died during follow-up) and was comparable with the mortality rate in patients without pathology of carbohydrate metabolism that was 27.3% (106 persons have died). Time of death after myocardial infarction also did not differ between the groups of working patients with DM and patients without DM, which is evidently demonstrated by the Kaplan-Meier curves (Figure 2).

### 3.3. Survival Rates of Patients after Myocardial Infarction Depending on the Diabetes Mellitus Duration

**Figure 3:**
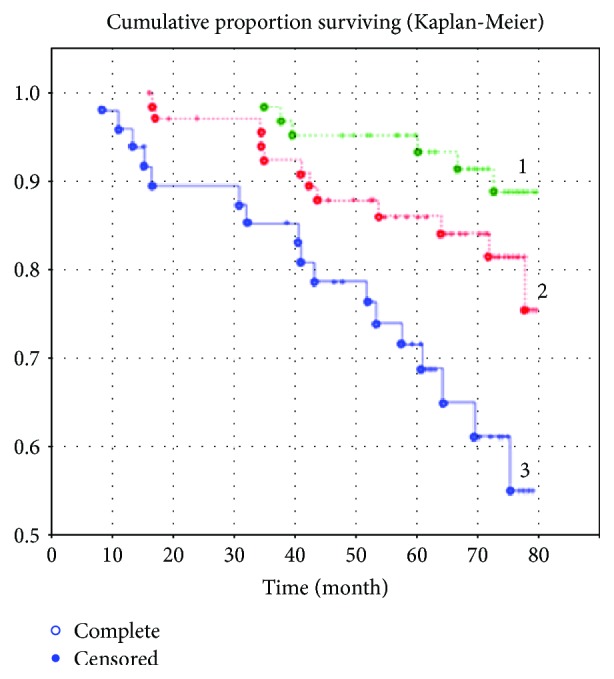
Survival rate of patients of working age with acute myocardial infarction according to duration of type 2 diabetes mellitus. Note: 1 = DM2 diagnosed in the postinfarction period (*n* = 38); 2 = DM2 diagnosed at the admission (*n* = 45); 3 = patients who had history of DM2 before the infarction occurred (*n* = 38).

In our study the comorbidities of type 2 DM and myocardial infarction (MI) in patients of working age did not show significant impact on long-term prognosis. For more detailed analysis, the group of working-age patients with both myocardial infarction and type 2 DM was assigned into 3 subgroups according to DM duration: the first subgroup included the patients that had longer duration of type 2 DM than cardiovascular disease, the second subgroup included patients with equal duration of both type 2 DM and cardiovascular disease, and the third subgroup included the patients with diabetes diagnosed during the five-year prospective study. Statistically significant differences in long-term outcomes were found in younger patients (*p* = 0.004) only in the group with longer duration of diabetes, unlike the group with DM2 onset (*p* = 0.004) (Figure 3).

## 4. Discussion

The results of the study showed that patients of the 1st group have significantly more serious complications due to their age, such as hypertension, angina, myocardial infarction(s), and stroke(s), which indicates the progression of existing diseases with age. At the same time, the disorder of lipid metabolism occurred with the same rate in both adult and older patients. Probably this indicator depends on genetic predisposition to the violation of lipid metabolism. In addition, the incidence of diabetes mellitus in both groups was the same at the time of inclusion of the patients into the study despite the fact that the duration of coronary artery disease was longer in the older patients. Possibly younger patients nowadays have lower physical activity and more refined food that contribute to the development of diabetes at younger age.

Our data suggest the generally accepted and proven hypothesis that type 2 diabetes mellitus increases the risk for adverse outcomes in elderly patients with MI. At the same time, we found that type 2 DM impact on long-term prognosis in patients of working age with MI strongly depends on duration of diabetes mellitus. In numerous studies, diabetes mellitus or hyperglycemia was shown to be an independent risk factor in both short- and long-term mortalities after acute MI [7]. Moreover, in the cohort study (VALIANT) diabetes mellitus was found to be an independent predictor of mortality and adverse cardiovascular events in patients of the first year after myocardial infarction. Analysis of these literature data has showed that the age of patients included in the studies was 60 years and older that corresponds to the patients of group 1 in our study which were older than the working age. Mechanisms of adverse outcomes among DM patients are related to impaired metabolic processes, including insufficiency of energy consumption of myocardium, activation of free radical processes, endothelial dysfunction, arterial thrombus formation, and fibrinolysis [8]. The DM patients are more vulnerable to development of atherosclerosis that leads to diffuse and multivascular damages of the coronary arteries. In addition, according to the literature data, diabetic cardiomyopathy can lead to the development of more severe forms of heart failure [8].

However, our study demonstrated that in the younger age group of the patients, the DM does not worsen prognosis of the survival rate but the survival rate depends on the duration of diabetes mellitus. So, the patients with DM developed after onset of myocardial infarction have better outcomes than the patients with DM diagnosed before or at the same time with myocardial infarction. But at the same time, some experimental studies show paradoxical increase of heart stability to damaging actions of ischemia and reperfusion in animals with DM [9, 10]. In previously published articles, we have also demonstrated that in inducing the onset of DM in rats in 2 weeks after myocardial infarction stimulation, the contractile function of isolated papillary muscle remains the same like in intact animals and the level of calcium-transporting proteins of sarcoplasmic reticulum of cardiomyocytes is also comparable with the one in healthy myocardium [11]. Moreover, we revealed that isolated trabeculae of the heart of ischemic heart disease (IHD) patients with short duration of DM maintained positive rhythm inotropic response of myocardium that correlated with a higher level of Са^2+^-ATPase of sarcoplasmic reticulum (SR) in comparison with the same parameters of myocardium in IHD patients without DM [12]. Since Са^2+^-ATPase of SR is energy-dependent enzyme, the efficiency of its work depends on not only the amount of enzyme but also on the availability of energy substrate. It has been shown that ATP produced in the glycolysis process is an essential source of energy for membrane transport of Ca^2+^, in particular for Са^2+^-ATPase ion pump of SR. In chronic ischemia, ATP synthesis in cardiomyocytes is carried out as a result of the process of glycolysis [13]. Probably in patients with CD2 at early stage of disease development, hyperglycemia can increase the availability of the additional substrates for glycolytic processes that promote normalization of energy supply of metabolic reactions in pathological cells. According to our previously published data at the combined development of postinfarction cardiosclerosis and DM in rats, cardiomyocytes use both fatty acids and glucose as energy substrate.

In accordance with our study, clinical data showing that patients with DM have the lower risk of adverse cardiovascular events in comparison with patients with ischemic heart disease has been appeared [14, 15]. In these studies, it is noted that mortality from acute myocardial infarction in patients with DM is lower than in patients with ischemic heart disease regardless of their age (the patients included in the study were 30–80 years).

However, type 2 diabetes of more than 10-year duration or type 2 DM with severely elevated glucose levels has been noted the same predictive value on the survival rate after myocardial infarction as for the IHD patients [14, 15]. In addition, a higher level of glucose in blood plasma at admission to hospital is an adverse prognostic factor for patients without diabetes in comparison with patients with established diagnosis of DM [16]. The presented data allow us to take a new look on the problem of DM in cardiovascular pathology and on the disclosure of the mechanisms causing the increase of heart resistance to ischemic damage. It is well known that all experimental works which shown the cardioprotective effect of DM were performed on young animals. That fact corresponds to our results obtained in group 2 [9, 10, 17, 18]. However, elderly Goto-Kakizaki rats (model of type 2 DM) have increased sensitivity to ischemic heart diseases [19].

Probably in the early developing of DM and in the patients of younger age, we can see the initiation of the so-called “metabolic preconditioning.” The mechanisms of this phenomenon can be connected with increase of the expression of antioxidant protection factors and enzymes of PI3K/Akt signaling pathways and with decrease of the expression of apoptosis genes, proinflammatory cytokines (TNF-*α*), profibrogenic transforming growth factor-*β*, and alpha actin-1—a hypertrophy marker [17]. Nowadays, the important role in realization of cardioprotective effects is given to protein—AMP-activated protein kinase—one of the key enzymes that initiates the process of energy saving in a cell. It was revealed that DM itself can lead to activation of this protein—AMP-kinase [20]; in addition, it was found that the expression rate of glucose transporter GLUT-1 in the cardiomyocytes of diabetic animals on 15th day after infarction was higher than the expression rate of glucose transporter in diabetic rats without ischemic damage [17]. Also, we can mention angiogenesis as a mechanism reducing the damage effects of ischemia before and after simulation of experimental myocardial infarction which is accompanied with decrease of the fibrosis processes in condition of hyperglycemia [17]. The sympathetic nervous system also plays a great role in pathogenesis of ischemic damages. It was found that while the sympathetic nervous system (SNS) tone decreases, induced by myocardial infarction in diabetic rats, the antioxidant protection of heart increases and of activity of prooxidant enzymes decreases [18]. Clinical data obtained from the study showed ambiguous prognostic effects of type 2 diabetes mellitus in patients with myocardial infarction consistent with those of experimental studies. Complex analysis of clinical and experimental data in this study area will help to develop the system of cardiovascular risk stratification in patients with both type 2 diabetes and coronary heart disease and will provide the opportunities for the personification of therapeutic approaches reducing damages of the ischemic factor effects.

## 5. Conclusions

The combination of both type 2 diabetes and coronary heart disease in elderly patients was showed to be a prognostically adverse factor, leading to a significant increase in the 5-year mortality rate. At the same time, younger patients with myocardial infarction did not have any significant differences in the long-term outcome according to the presence of diabetes mellitus while a longer duration of DM2 significantly worsened prognosis during the postinfarction period.

## Figures and Tables

**Table 1:** Clinical characteristics of the patients with myocardial infarction in dependence of the age.

Indicators	The 1st group (*n* = 358)	The 2nd group (*n* = 504)	*p*
Men/women, *n* (%)	158/200 (44/56)	433/71 (86/14)	<0.001
Infarction, *n* (%)	122 (34)	85 (17)	<0.001
Angina, *n* (%)	255 (71.2)	204 (40)	<0.001
Stroke, *n* (%)	46 (12.8)	36 (7)	0.005
Arterial hypertension, *n* (%)	320 (89.3)	323 (64)	<0.001
Dyslipidemia, *n* (%)	283 (79)	386 (76.6)	0.4
Smoker, *n* (%)	113 (31.6)	403 (80)	<0.001
Presence of type 2 diabetes mellitus, *n* (%)	92 (26)	116 (23)	0.36
Glycemic level, *μ* ± *σ*	6.49 ± 1,9	5.99 ± 1,93	0.0002
Atypical level of infarct, *n* (%)	15 (4.2)	64 (12.7)	<0.001
Infarction with ST elevation, *n* (%)	279 (78)	403 (80)	0.47

Note: *μ* ± *σ*: mean value and mean square deviation; *р*: achieved significant level.

## Data Availability

The data used to support the findings of this study are available from the corresponding author upon request.
