# Corrigendum to “The Impact of Type 2 Diabetes Mellitus on Long-Term Prognosis in Patients of Different Ages with Myocardial Infarction”

**DOI:** 10.1155/2019/8347891

**Published:** 2019-03-04

**Authors:** S. A. Afanasiev, A. A. Garganeeva, E. A. Kuzheleva, A. V. Andriyanova, D. S. Kondratieva, S. V. Popov

**Affiliations:** Cardiology Research Institute, Tomsk NRMC, 111a Kievskaya Street, Tomsk 634012, Russia

In the article titled “The Impact of Type 2 Diabetes Mellitus on Long-Term Prognosis in Patients of Different Ages with Myocardial Infarction” [1], the authors' affiliation was not accurately named as “Tomsk NRMC” was missing from it. The corrected affiliation is shown above.
